# Comprehensive analysis on the expression levels and prognostic values of LOX family genes in kidney renal clear cell carcinoma

**DOI:** 10.1002/cam4.3472

**Published:** 2020-09-24

**Authors:** Shitong Lin, Lingling Zheng, Yuchao Lu, Qidong Xia, Peng Zhou, Zheng Liu

**Affiliations:** ^1^ Cancer Biology Research Center (Key Laboratory of the Ministry of Education) Tongji Hospital Tongji Medical College Huazhong University of Science and Technology Wuhan China; ^2^ Department of Nuclear Medicine Huashan Hospital Fudan University Shanghai China; ^3^ Department of Urology Tongji Hospital Tongji Medical College Huazhong University of Science and Technology Wuhan China

**Keywords:** extracellular matrix, kidney renal clear cell carcinoma, LOX, LOX family genes, LOXL2

## Abstract

**Backgrounds:**

Kidney renal clear cell carcinoma (KIRC) is a major pathological type of renal cell carcinoma (RCC), and the prognosis of advanced KIRC patients is often unsatisfactory. Some lysine oxidase (LOX) family genes have been proven to be upregulated in some malignancies and play pivotal roles in the carcinogenesis. However, their roles in KIRC remain unclear.

**Materials and Methods:**

Here, we used some online databases (eg, ONCOMINE, GEPIA, UALCAN, c‐BioPortal, Human Protein Altas) to comprehensively explored the expression levels and the prognostic values of LOX family genes in KIRC using bioinformatic methods.

**Results:**

The results revealed that lysyl oxidase (LOX) and lysyl oxidase‐like 2 (LOXL2) were significantly overexpressed in KIRC at the level of mRNA expression, protein expression, and RCC cell lines. Further analysis demonstrated that higher mRNA expression of *LOX* and *LOXL2* were significantly correlated with poor survival, tumor grade, individual cancer stages, and nodal metastasis status. DNA copy number amplifications and mRNA upregulation, DNA deep deletion, and mRNA upregulation were the main genetic mutations of *LOX* and *LOXL2*, respectively. Prognostic analysis showed that the altered group had significantly poorer overall survival (OS) compared to the unaltered group (*p = *.0387). Co‐expression analysis showed *CP*, *PLOD2*, and *COL5A1* were significantly correlated with *LOX*, and *COL1A2* was positively correlated with *LOXL2*. Further analysis confirmed that these co‐expressed genes were significantly upregulated and predicted unfavorable prognosis in KIRC.

**Conclusion:**

Multi‐level analysis demonstrated that *LOX* and *LOXL2* were significantly upregulated and predicted poor survival in KIRC, which may apply as promising biomarkers for diagnosis and therapy of KIRC in the future.

## Graphical abstract

We firstly comprehensively explored the role of LOX family genes in kidney renal clear cell carcinoma, and found that LOX and LOXL2 may be used as new therapeutic targets for kidney renal clear cell carcinoma.

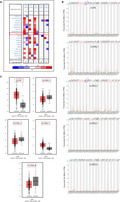

## 1 INTRODUCTION

Renal cell carcinoma (RCC), also known as renal adenocarcinoma, is one of the most common malignant tumors in the kidney.^1^ It was estimated that there were 403,262 new cases and 175,098 deaths in 185 countries according to global cancer statistics 2018.^2^ KIRC is the main pathological type of RCC, which accounting for 80%.^3^ The 5‐year overall survival rate (OS) of early stage KIRC could reach 96%, but it is no more than 10% for advanced stages.^1^ In addition to surgical resection, chemotherapy, and radiotherapy were once the main adjuvant therapy after surgery.^4^, ^5^ However, with the development of medical research, targeted therapy combined with immunotherapy has gradually become the main treatment for postoperative RCC patients, especially for advanced stage disease and relapsed patients.^6^, ^7^ Therefore, it is crucial to explore genomic mutation characteristics and identify biomarkers of RCC, which will make contributions to the early diagnosis and targeted therapy of RCC.

The LOX family gene is a class of genes encoding copper dependent amine oxidase, which plays pivotal role in catalyzing the oxidative deamination of E‐amino group in collagen and elastin to promote their crosslinking in extracellular matrix (ECM), thereby enhancing the structural integrity and tensile strength of connective tissue.^8^, ^9^, ^10^ It consists of five members, namely lysyl oxidase (*LOX*) and four lysyl oxidase‐like genes (lysyl oxidase‐like 1, *LOXL1*; lysyl oxidase‐like 2, *LOXL2*; lysyl oxidase‐like 3, *LOXL3*; and lysyl oxidase‐like 4, *LOXL4*) respectively.^11^, ^12^, ^13^, ^14^ Researchers found that some LOX family genes are differently expressed and are involved in the occurrence and development of certain malignancies. It has been further noted that these genes are significantly related to patient prognosis. Hiroaki Kasashima et al found LOXL1, LOXL3, and LOXL4 are significantly correlated with distant metastasis in gastric cancer.^15^ Cao Canhui et al found that over‐expression of *LOXL2* significantly predicts poor survival in cervical cancer via remolding epithelial‐mesenchymal transition (EMT).^16^

Before carrying out the research, we have found that some LOX family genes, such as *LOX* and *LOXL2*, were significantly correlated with poor prognosis in many types of solid tumors (eg, bladder urothelial carcinoma, lung squamous cell carcinoma, and skin cutaneous carcinoma) using the online web tool ESurv, which was a user‐friendly web tool for survival analysis.^17^ Here, we aimed to comprehensively explore the expression level, prognostic values, and mutation characteristics of LOX family genes in KIRC, and identify promising biomarkers that may play key roles in the occurrence and development of KIRC.

## 2 MATERIALS AND METHODS

### 2.1 Oncomine database analysis

Oncomine (https://www.oncomine.org/resource/login.html#) is the world's largest cancer gene chip database and integrated database mining platform. At present, 729 gene expression data sets, more than 90,000 cancer tissue and normal tissue sample data have been collected. Oncomine can be used to compare the different expression of cancer types and their normal expression tissues.^18^ We used the Oncomine database to determine the expression levels of LOX family genes in human cancers, especially in RCC. We also performed meta‐analyses on related KIRC studies to further confirm LOXs expression levels. The thresholds were as follows: fold change >1.5, *p* < .05, and the top gene rank was 10%.

### 2.2 GEPIA analysis

The online database Gene Expression Profiling Interactive Analysis (GEPIA) (http://gepia.cancer‐pku.cn/detail.php) is an open public database, which contains the expression data of 9736 tumors and 8587 normal samples from TCGA and GTEX projects. The expression data of TCGA and GTEX are recalculated under the same pipeline, and can be directly analyzed.^19^ First, we used it to explore the expression levels of *LOX* and *LOXL2* in KIRC, kidney renal papillary cell carcinoma (KIRP), and kidney chromophobe (KICH). Second, we used it to measure the relationships between LOXs and 10 hub genes using Pearson's Correlation Coefficient. Finally, we used it to explore the prognostic values of *LOX* and *LOXL2* in KIRP and KICH.

### 2.3 UALCAN analysis

UALCAN (http://manualcan.path.uab.edu/index.html) is an effective website for online analysis and mining of cancer data, mainly based on the analysis of relevant cancer data in TCGA database. It aids medical researchers to identify the relevant genes, analyze the expression spectrum, analyze the survival, etc, and also query the relevant information in other databases through relevant links.^20^ We used this database to explore the prognostic values of *LOX* and *LOXL2* in KIRC, and further measured the relationships between their mRNA transcriptional levels and clinicopathological parameters in KIRC.

### 2.4 c‐BioPortal database analysis

The c‐Bio Cancer Genomics Portal (c‐BioPortal) (https://www.cbioportal.org/) integrates and simplifies the contents of several cancer genome databases including TCGA, ICGC, and Geo. It mainly shows the somatic mutation spectrum, copy number change, mRNA miRNA expression change, DNA methylation, and protein expression of the gene. Combined with the clinical data of patients, it shows the survival curve of KM.^21^ We used it mainly to obtain mutation data of *LOX*, and *LOXL2* in KIRC. We also explored their prognostic values in altered KIRC patients. Co‐expressed genes of *LOX* and *LOXL2* were identified by the column of “Co‐expression” in c‐BioPortal.

### 2.5 TIMER analysis

TIMER (https://cistrome.shinyapps.io/timer/) is an online database that can be used to systematically analyze the immune infiltration status of various cancer types. It uses the TIMER algorithm to estimate the abundance of six immune infiltration fluids (B cells, CD4+ T cells, CD8+ T cells, neutrophils, macrophages, and dendritic cells) in various cancers.^22^, ^23^ In our research, we mainly use it to explore the relationship between LOX, LOXL2, and immune infiltration.

### 2.6 Functional enrichment analysis

Metascape (http://metascape.org) is an easy‐to‐operate online web tool that can be used for gene annotation and analysis to help biologists understand one or more gene lists. Metascape provides automated meta‐analysis tools to understand a set of common and unique approaches in orthogonal target discovery research.^24^ In this study, we used it to perform enrichment analysis of *LOX*, *LOXL2* and their identified co‐expressed genes.

### 2.7 Identification of hub genes of LOX and LOXL2

Cytoscape is a software that focuses on open source network visualization and analysis. Its core is to provide the basic function layout and query network, and to combine the basic data into a visual network. We can integrate these biological networks with gene expression, genotype, and other molecular state information in a visual environment, and link these networks with functional annotation database through Cytoscape.^25^ In our study, hub genes of LOXs and their interaction networks were identified according to the degree scores using cytoHubba tool kits in Cytoscape.

### 2.8 HPA database analysis

The Human Protein Altas (HPA) (https://www.proteinatlas.org/) provides information on the tissue and cell distribution of all 24,000 human proteins. It uses special antibodies and immunohistochemistry technology to check the distribution and expression of each protein in 48 kinds of normal human tissues, 20 kinds of tumor tissues, 47 cell lines and 12 kinds of blood cells. These tissues come from 144 different individuals and 216 tumor tissues, which ensure that the staining results are fully representative.^26^ We used this database to confirm prognostic significance and protein expression levels of the most potential hub genes in KIRC.

## 3 RESULTS

### 3.1 Over‐expression levels of *LOX* and *LOXL2* at the level of mRNA, protein, and cancer cell lines in RCC

**FIGURE 1:**
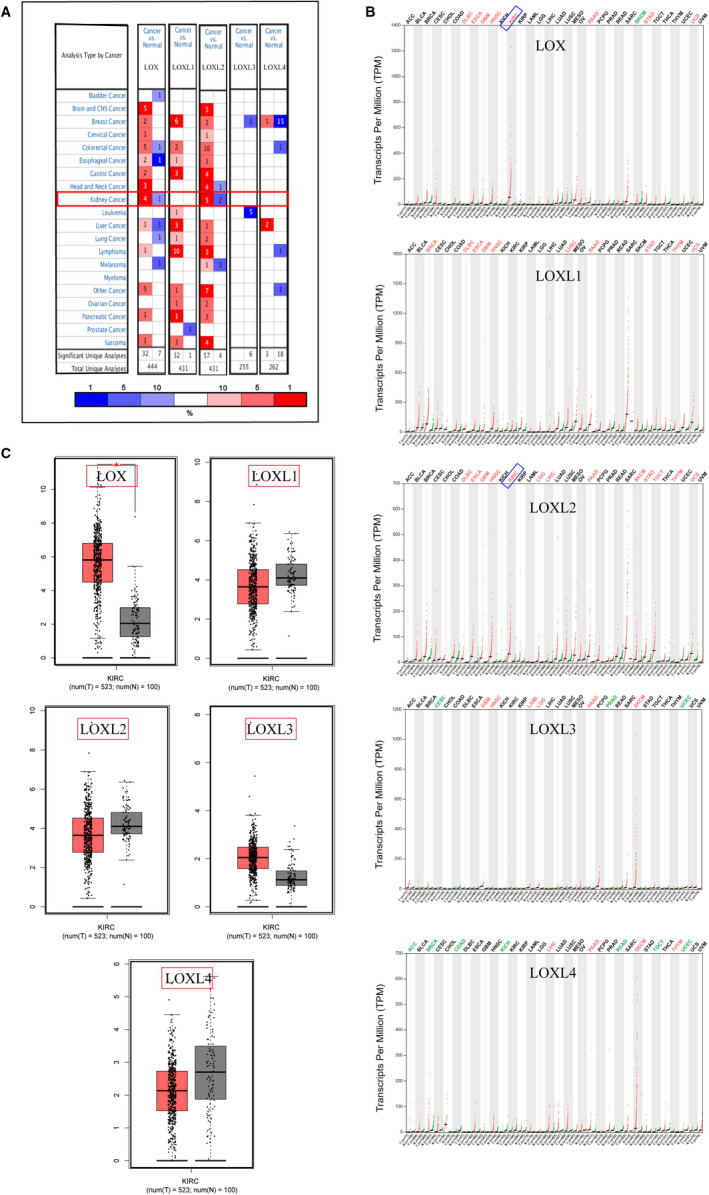
Upregulated mRNA transcriptional levels of *LOX* and *LOXL2* in KIRC. A, The numbers in each table represented the studies with statistically significant tumor tissue mRNA over‐expression (red) or down‐expression (blue) in Oncomine, and *LOX* and *LOXL2* were significantly upregulated in kidney cancer. B,C, Overexpressed expression levels of LOX and LO*XL2* at the level of tumor tissue mRNA (n = 523) compared to normal tissues in KIRC (n = 100) were further confirmed using GEPIA

**FIGURE 2:**
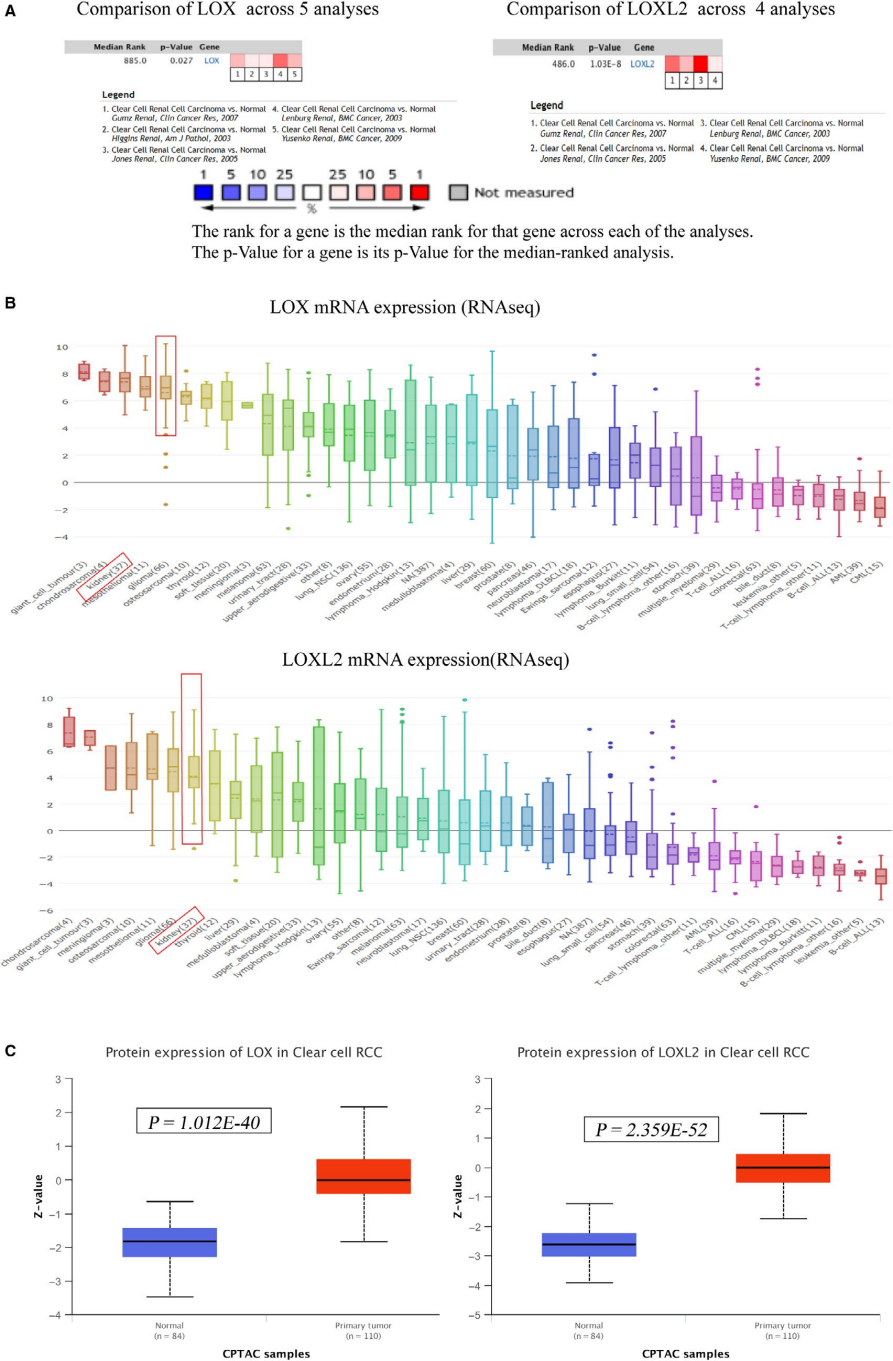
Overexpression levels of *LOX* and *LOXL2* at the level of KIRC cell lines and tumor tissue protein. A, Meta‐analyses of related studies in KIRC demonstrated *LOX* and *LOXL2* were significantly upregulated in tumor tissues compared to normal tissues using the Oncomine. B, *LOX* and *LOXL2* mRNA expression levels in kidney cancer cell lines were higher than most other human cancer types using the CCLE. C, Protein expression levels of *LOX* and *LOXL2* in KIRC tumor tissues were significantly overexpressed than normal tissues in CPTAC samples using the UALCAN

We used the online database ONCOMINE to explore the expression profiles of LOX family genes in certain human cancers. As shown in Figure 1A, *LOX*, *LOXL1*, and *LOXL2* were upregulated in certain kinds of cancers, and only *LOX* and *LOXL2* were significantly elevated in RCC. These findings were further confirmed by other online databases at the level of mRNA, protein, and RCC cell lines. As shown in Figure 1B,C, *LOX* and *LOXL2* were significantly over‐expressed in KIRC tumor compared to normal tissue using GEPIA. Meta‐ analysis of related KIRC studies contained in ONCOMINE were also consistent with the above findings (Figure 2A). We also used CCLE to explore their expression levels in RCC cell lines using the broad institute cancer cell line encyclopedia (CCLE). As shown in Figure 2B, *LOX* and *LOXL2* were significantly upregulated in RCC cell lines than other cancers. More importantly, protein expression levels of LOX (*p* = 1.012E‐ 40) and LOXL2 (*p* = 2.359E‐52) were also significantly elevated in CPTAC samples (normal = 80, primary tumor = 110) using UACLAN (Figure 2C). We also further explored the expression levels of *LOX* and *LOXL2* in KIRP and KICH. As shown in Figure S1A,B, neither *LOX* nor *LOXL2* was differentially expressed in KICH or KIRP tissues compared to normal tissues. Collectively, all these findings strongly confirmed the high expression status of *LOX* and *LOXL2* in KIRC.

### 3.2 Upregulation of *LOX* and *LOXL2* were significantly related to poor survival and clinicopathological parameters in KIRC

**FIGURE 3:**
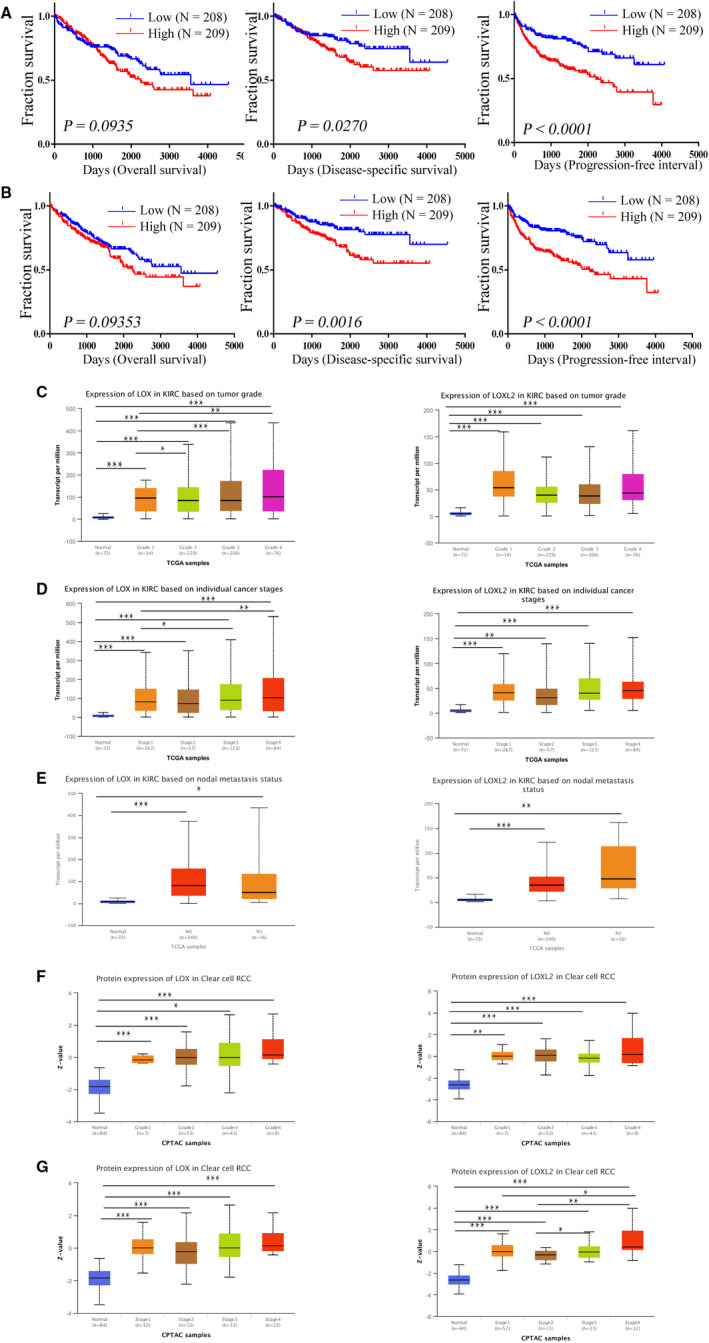
Relationship between *LOX* and *LOXL2* mRNA expression levels and patients’ survival and clinicopathological characteristics in KIRC using the UALCAN. A, Over‐expression of *LOX* significantly correlated with poor disease‐specific survival (DSS) and progression‐free survival (PFS) in KIRC. B, Upregulated *LOXL2* also predicted poor DSS and PFS in KIRC. D‐F, higher mRNA expression of *LOX* and *LOXL2* were significantly correlated with tumor grade, individual cancer stages and nodal metastasis status in KIRC, respectively. G,H, over‐expressed protein expression levels of *LOX* and *LOXL2* were significantly closed to tumor grade and individual cancer stages. *p < .05; **p < .01; ***p < .001

We downloaded the expression levels of LOX and LOXL2 of TCGA patients from c‐BioPortal, and obtained clinical data of TCGA (KIRC) from an integrated clinical data resource, which provide an unprecedented sale to perform high‐quality prognostic analysis.^21^, ^27^ As shown in Figure 3A,B, Over‐expression of LOX significantly correlated with poor disease‐specific survival (DSS) (*p* = .0270) and progression‐free survival (PFS) (*p* < .0001) in KIRC, and upregulated LOXL2 also predicted poor DSS (*p* = .0016) and PFS (*p* < .0001) in KIRC. We then further explored the significance of the higher expression of *LOX* and *LOXL2* regarding clinicopathological parameters in KIRC using UALCAN. As shown in Figure 3C,D,E, overexpressed mRNA of *LOX* and *LOXL2* were significantly correlated with tumor grade, individual cancer stages and nodal metastasis status in KIRC from TCGA samples. Tumor grade and individual cancer stages in KIRC were also significantly affected by higher protein expression of *LOX* and *LOXL2* from CPTAC samples (Figure 3F,G). We also explored the prognostic values of *LOX* and *LOXL2* in KIRP and KICH. As shown in Figure S1C,D, higher expression of *LOX* was significantly correlated with poor OS (HR = 9.3, *p* = .035) and disease‐free survival (HR = 11, *p* = .023) in KICH, and upregulated *LOX* predicted poor OS in KIRP (HR = 2.3, *p* = .012), respectively (Figure S1C). Over‐expression of *LOXL2* significantly predicted poor disease‐free survival in KICH (HR = 9.5, *p* = .033) (Figure S1D).

### 3.3 Genetic mutations analysis of LOXs and their association with poor OS in KIRC

**FIGURE 4:**
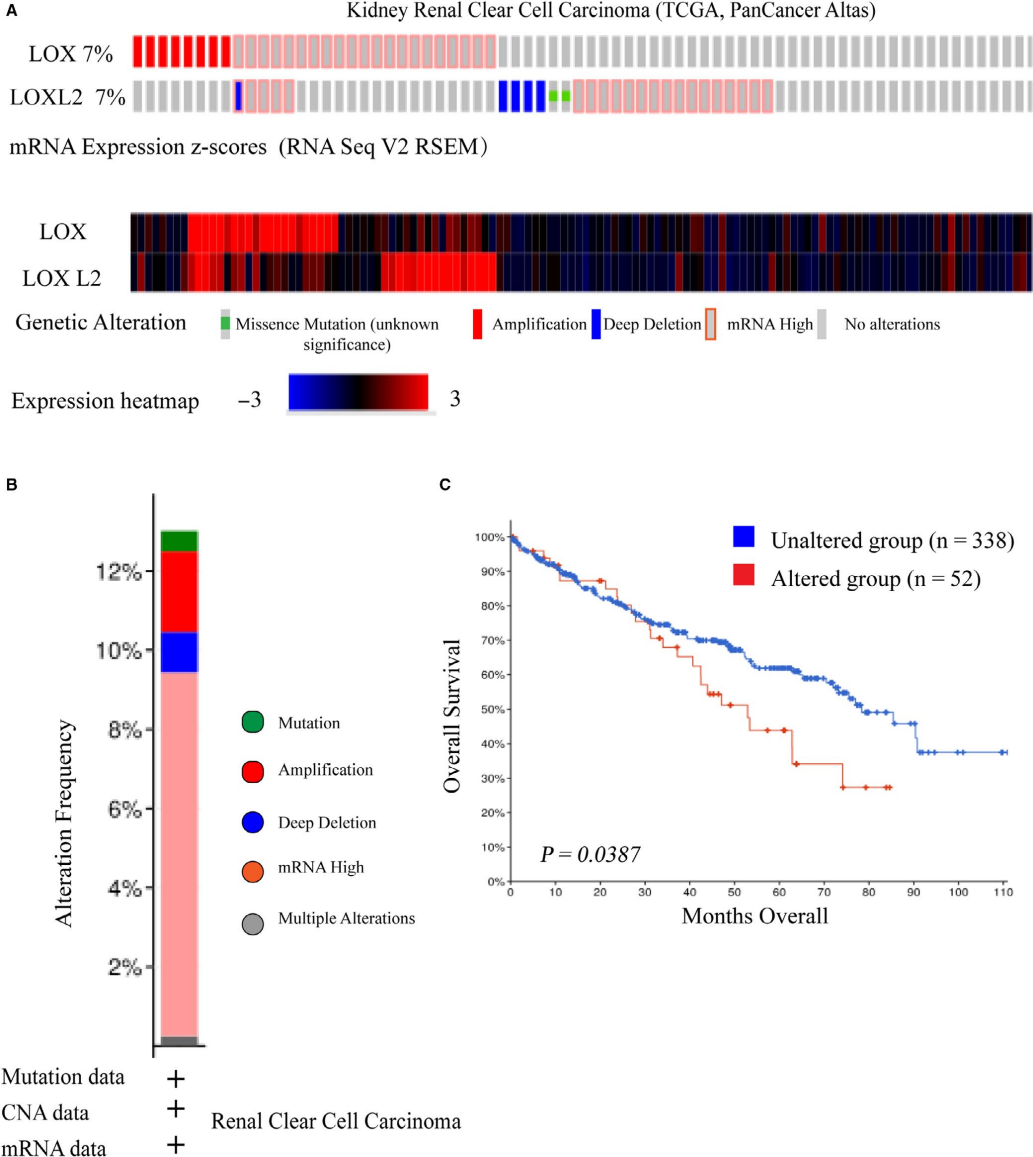
Genetic mutations and their association with KIRC prognosis of *LOX and LOXL2*. A, OncoPrint of c‐BioPortal showed the mutation types and proportions of *LOX* and *LXOL2* from TCGA samples, respectively. B, Cancer types summary of c‐BioPortal displayed the types of mutations and their proportions contained in each cancer type of this selected study (TCGA, Firehose Legacy). C, Higher mutations of *LOX* plus *LOXL2* significantly predicted poor overall survival in KIRC

We also explored mutation characteristics of *LOX* and *LXOL2* in KIRC using c‐BioPortal. As shown in Figure 4A, *LOX* and *LOXL2* shared the same high mutation frequencies (7%) in KIRC (TCGA, PanCancer Atlas). DNA copy number amplifications and mRNA upregulation, DNA deep deletion and mRNA upregulation were the main genetic mutations of *LOX* and *LOXL2*, respectively (Figure 4B). Further analysis revealed that altered group (n = 52) shared a significantly poor OS compared to unaltered group (n = 52) (Figure 4C).

### 3.4 Correlation analysis of *LOX* and *LOXL2* expression and immune infiltration of immune cells in KIRC using the TIMER database

**FIGURE 5:**
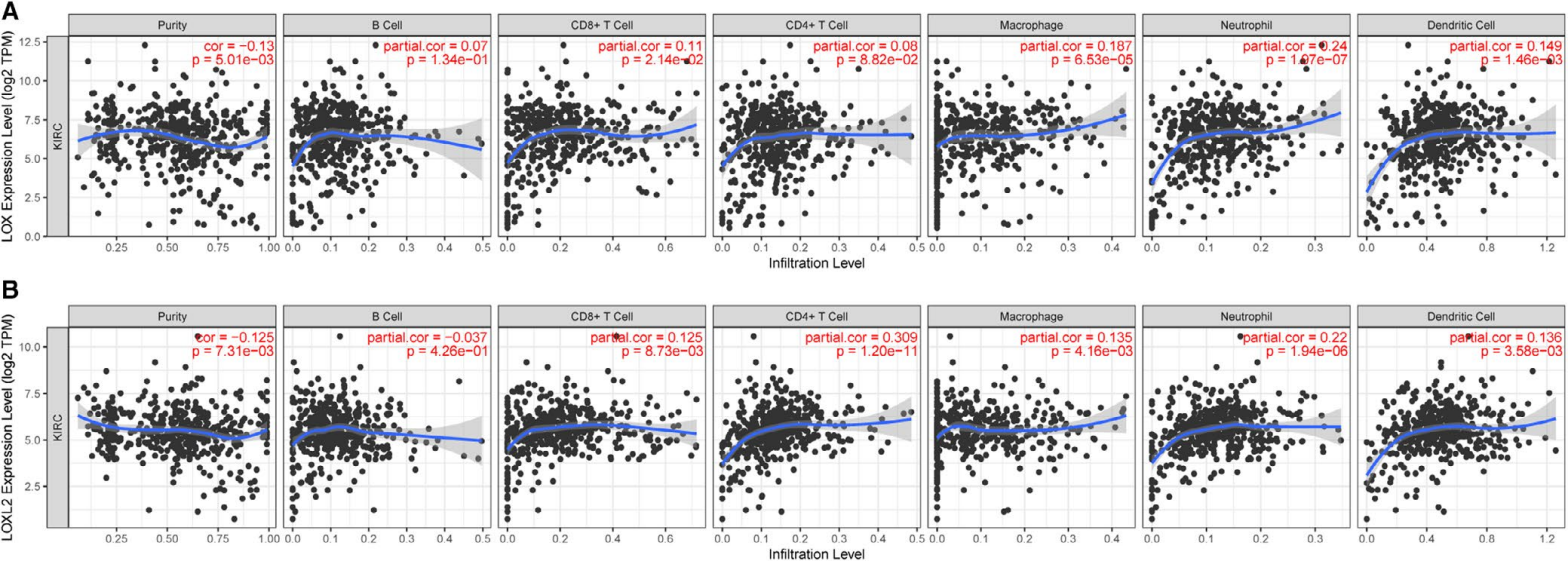
Correlation analysis of *LOX* and *LOXL2* expression and immune infiltration of immune cells in KIRC using the TIMER database. A, *LOX* expression positively correlated with CD8+ T cell, Macrophage, Neutrophil, and Dendritic cell in KIRC tissues. (B) *LOXL2* significantly correlated with CD8+ T cell, CD4+ T cell, Macrophage, Neutrophil, and Dendritic cell in KIRC tissues

It was reported that the number and activity of tumor infiltrating lymphocytes could significantly affect the prognosis of cancers. Here, we aimed to explore the relationships between the expression levels of *LOX* and *LOXL2* and immune infiltration of immune cells. As shown in Figure 5A, *LOX* was positively corelated with CD8+ T cell (partial. Cor = 0.11, *p* = 2.14E‐02), Macrophage (partial. Cor = 0.187, *p* = 6.53E‐05), Neutrophil (partial. Cor = 0.24, *p* = 1.97E‐07), and Dendritic cell (partial. Cor = 0.149, *p* = 1.46E‐03) in KIRC *LOXL2* was positively correlated with CD8+ T cell (partial. Cor = 0.125, *p* = 8.73E‐03), CD4+ T cell (partial. Cor = 0.309, *p* = 1.20E‐11), Macrophage (partial. Cor = 0.135, *p* = 4.16E‐03), Neutrophil (partial. Cor = 0.22, *p* = 1.94E‐06), and Dendritic cell (partial. Cor = 0.136, *p* = 3.58E‐03) (Figure 5B).

### 3.5 Enrichment analysis of genes that positively correlated with *LOX* and *LOXL2*

**FIGURE 6:**
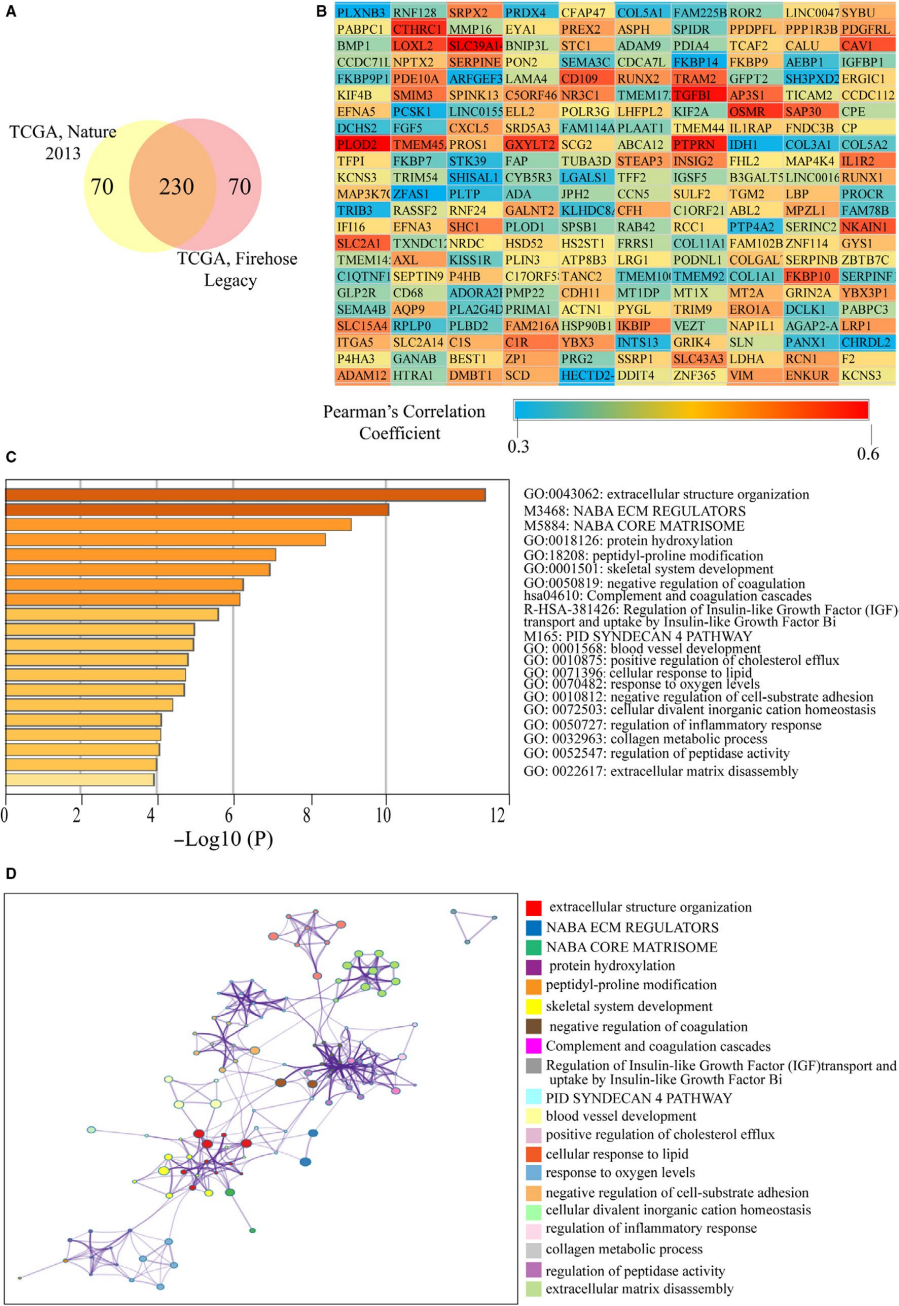
Functional enrichment analysis of co‐expressed genes of *LOX*. A, The Venn diagram represents the 230 genes co‐expressed with *LOX* found in two other TCGA studies on KIRC. B, The heat map represents the correlation between LOX and its 230 co‐expressed genes according to the values of Pearson Correlation Coefficient. C, Heatmap of enriched terms regarding Gene Ontology across *LOX* and its co‐expressed genes constructed by Metascape. D, Interactive network of the top 20 enriched terms colored by cluster ID. Each color represents one enrichment pathway

**FIGURE 7:**
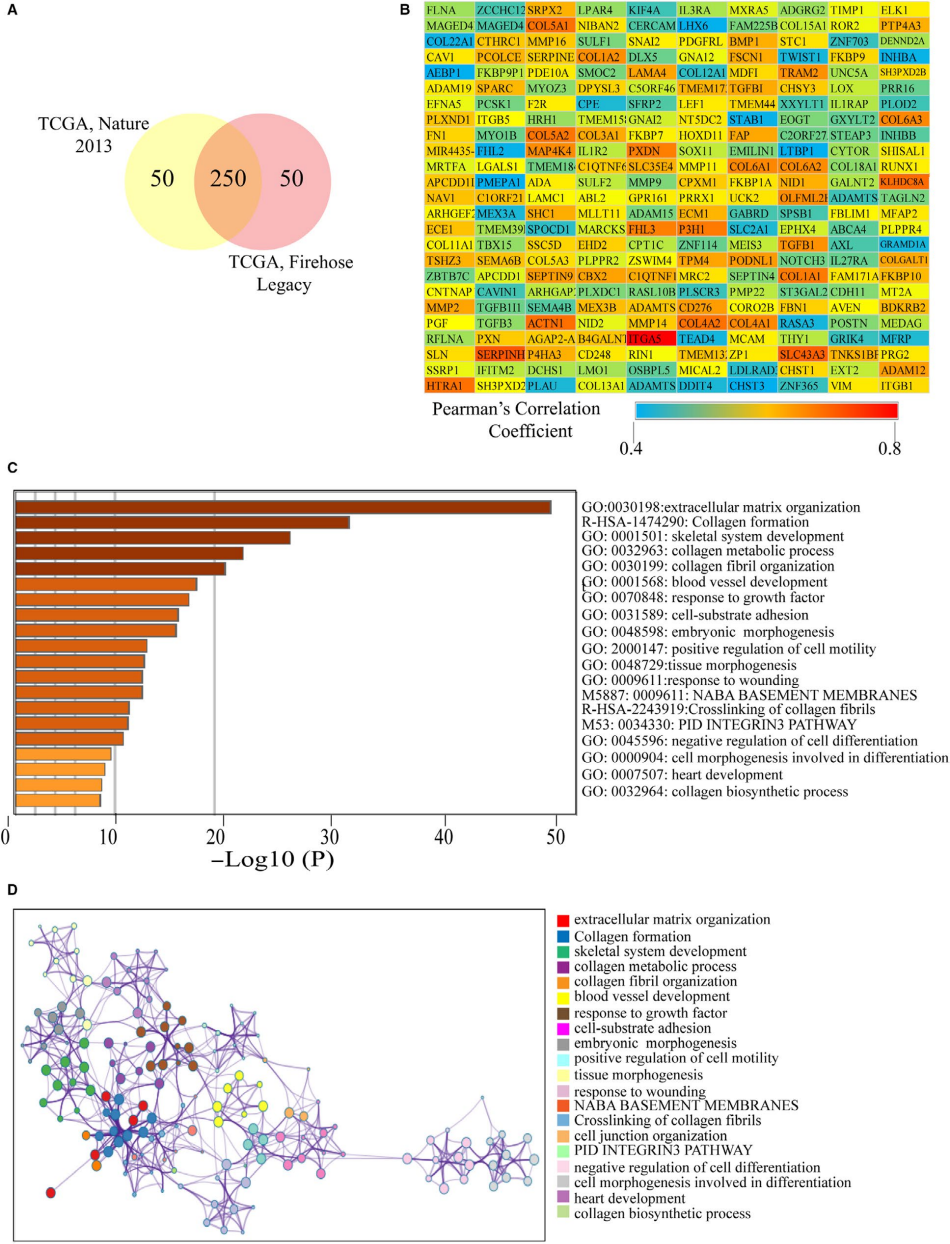
Enrichment analysis of co‐expressed genes of *LOXL2*. A, The Venn diagram represents the 230 genes co‐expressed with *LOXL2* found in two other TCGA studies on KIRC. B, The heat map represents the correlation between LOXL2 and its 250 co‐expressed genes according to the values of Pearson Correlation Coefficient. C, Heatmap of enriched terms regarding Gene Ontology across *LOXL2* and its co‐expressed genes constructed by Metascape. D, Interactive network of the top 20 enriched terms colored by cluster ID. Each color represents one enrichment pathway

The c‐BioPortal was used to identify top 300 co‐expressed genes with *LOX* and *LXOL2* in two different studies from TCGA (TCGA, Nature 2013; TCGA, Firehorse Legacy). As shown in Figures 6A and 7A, *LOX* and *LOXL2* had 230 and 250 positively co‐expressed genes, which were duplicate genes in two TCGA studies. The values of Spearman's correlation were shown in Figures 6B and 7B. In order to further explore enrichment function of co‐expressed genes, we performed the analyses of Gene Ontology (GO) and Kyoto Encyclopedia of Genes and Genomes (KEGG) pathway using the Metascape. As shown in Figure 6C, co‐expressed genes of *LOX* were significantly correlated with extracellular structure organization, and got involved in the pathway of NABA ECM REGULATORS. *LOXL2* and its co‐expressed genes were closely correlated with extracellular matrix organization, skeletal system development, and collagen metabolic process, and they were got involved in the pathway of collagen formation and crosslinking of collage fibrils (Figure 7C). The networks of enrichment terms of *LOX* and *LOXL2* according by cluster ID were displayed in Figures 6D and 7D. In summary, these results indicated that *LOX* and *LOXL2* may play pivotal roles in KIRC through mediating the formation of ECM.

### 3.6 *LOX* and *LOXL2* PPI networks construction and identification of hub genes

**FIGURE 8:**
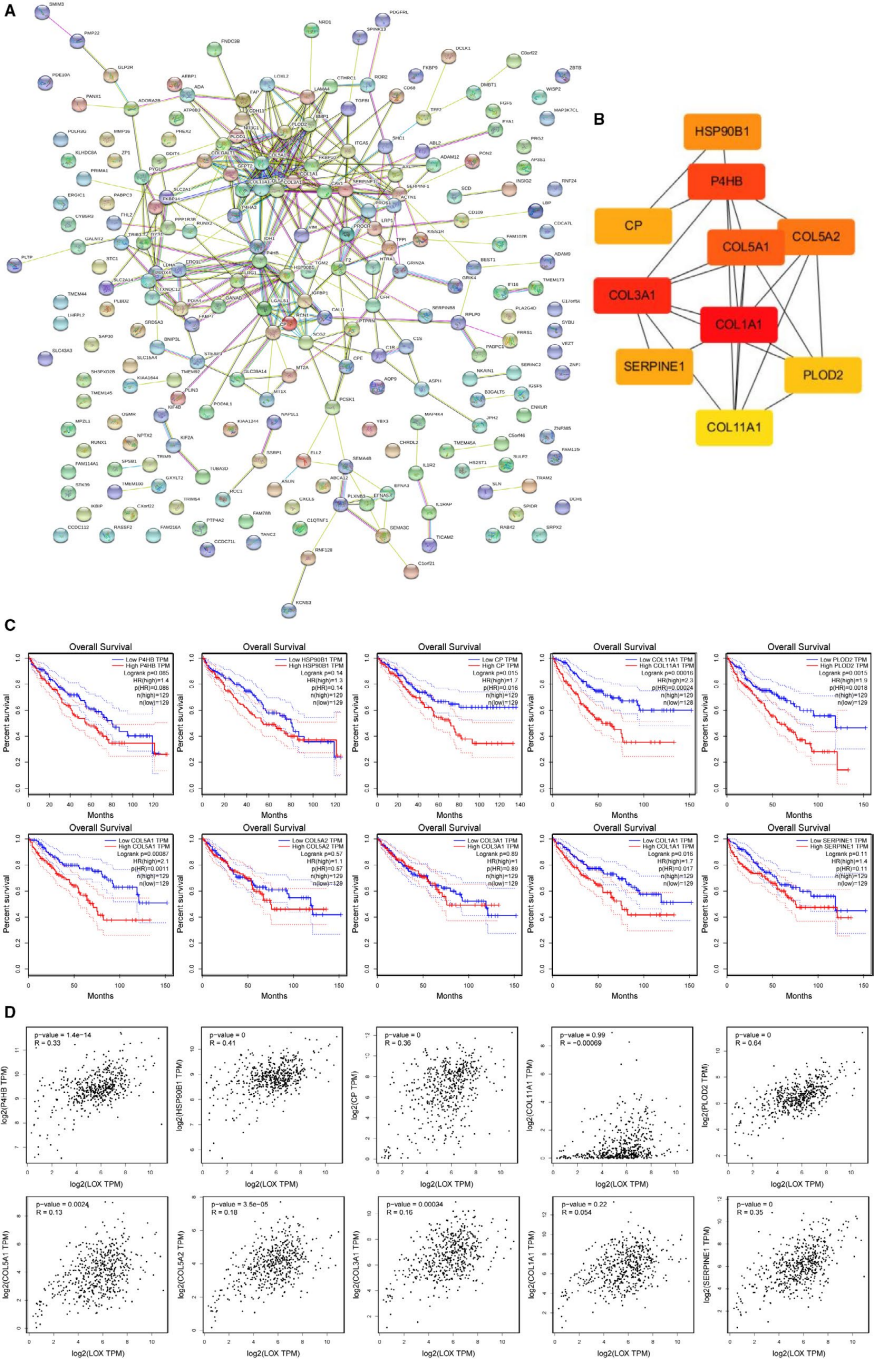
Identification of hub genes among the co‐expressed genes of *LOX*. A, protein‐protein interaction network of 230 co‐expressed genes of LOX constructed by STRING. B, Ten hub genes of *LOX* were identified using cytoHubba tool kits in Cytoscape. C, Overall survival analyses of 10 hub genes in KIRC using the GEPIA among TCGA patients. D, Correlations between *LOX* and 10 hub genes mRNA expression determined using the GEPIA

**FIGURE 9:**
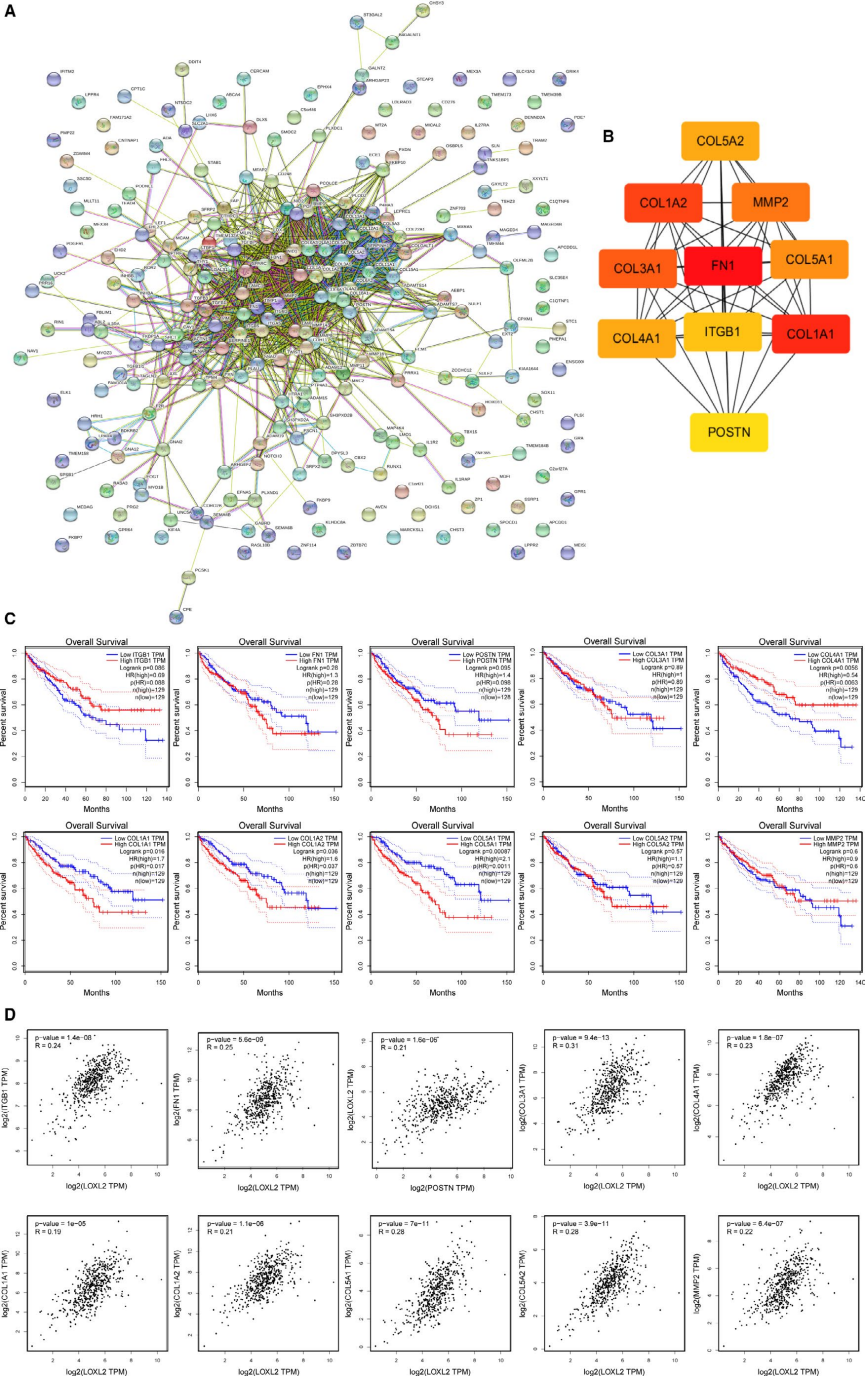
Identification of hub genes among the co‐expressed genes of *LOXL2*. A, protein‐protein interaction network of 250 co‐expressed genes of *LOX*L2 constructed by STRING. B, Ten hub genes of *LOXL2* were identified using cytoHubba tool kits in Cytoscape. C, Overall survival analyses of 10 hub genes in KIRC using the GEPIA among TCGA patients. D, Correlations between *LOXL2* and 10 hub genes mRNA expression determined using GEPIA

230 co‐expressed genes of *LOX* and 250 co‐expressed genes of *LOXL2* were imported into STRING to construct their own PPIs (Figures 8A and 9A). The top 10 hub genes of each PPI were identified according to the degree scores using cytoHubba tool kits in Cytoscape. As shown in Figure 8B, *SERPINE1*, *COL1A1*, *P4HB*, *CP*, *HSP90B1*, *COL5A1*, *COL5A2*, *PLOD2*, *COL3A1*, and *COL11A1*were the top 10 hub genes of *LOX*. *MMP2*, *COL1A1*, *COL4A1*, *COL5A2*, *COL3A1*, *COL1A2*, *FN1*, *POSTN*, *ITGB1*, and *COL5A1* were the top 10 hub genes of *LOXL2* (Figure 9B). We then further measured their prognostic values in KIRC and relationships with *LOX* and *LOXL2* using the GEPIA among TCGA patients. As shown in Figure 8C,D, over‐expression of *CP* (HR = 1.7, *p* = .016), *COL11A1* (HR = 2.3, *p* = .00024), *PLOD2* (HR = 1.9, *p = *.0018), *COL5A1* (HR = 2.1, *p = *.0011), and *COL1A1* (HR = 1.7, *p = *.017) were positively correlated with poor OS in KIRC *CP* (*R* = .36, *p* = 0) and *PLOD2* (*R* = .64, *p* = 0) were selected as the most potential hub genes of *LOX* after excluding genes with *R* value of Pearson's correlation <.2. After the same process, *COL1A2* (HR = 1.6, *p* = .037; *R* = 0.21, *p* = 1.1E‐06) was considered as the most potential hub gene of *LOXL2* (Figure 9C,D). All these findings indicated that these potential hub genes may play pivotal roles in KIRC by cooperating with *LOX* and *LOXL2*.

### 3.7 Prognostic values and protein expression levels of hub genes in KIRC from TCGA samples

We further confirmed prognostic values and protein expression levels of these selected hub genes in KIRC using the HPA. As shown in Figure S2A, high expression levels of *CP* (*p* = 1.1E‐07), *PLOD2* (*p* = 5.0E‐11), and *COL1A2* (*p* = 1.5E‐09) significantly correlated with poor overall survival in KIRC, respectively. Protein expression levels of these selected hub genes in KIRC were validated using immunohistochemical staining (IHF) from HPA (Figure S2B). All these findings demonstrated that these co‐expressed hub genes may play essential role in KIRC by cooperating with *LOX* and *LOXL2*.

## 4 DISCUSSION

KIRC is the most common type of RCC, but its prognosis is poor. For postoperative KIRC patients, recurrence or distant metastasis occurs in 30% of patients. Therefore, it is of great significance to identify unique therapeutic targets of KIRC to improve the prognosis of patients.^28^ In recent years, molecular biology technology and bioinformatics have rapidly developed. Some potential RCC therapeutic targets have been identified using bioinformatics, such as ALOX5, METTL14, et al.^29^, ^30^

For the first time, we used multiple online databases to comprehensively investigate the expression levels of the LOX family genes in KIRC at the levels of tumor tissue mRNA, tumor tissue protein and RCC cell lines mRNA, and further explored their significance on prognosis and molecular mechanism in KIRC. LOX family genes have been shown to play key roles in the formation and remodeling of ECM, which could affect biological processes such as cell differentiation, proliferation, adhesion, morphogenesis and phenotypic expression.^31^, ^32^ Finally, we found that *LOX* and *LOXL2* were significantly upregulated and predicted poor survival in KIRC. Furthermore, enrichment analyses of *LOX*, *LOXL2* and their co‐expressed genes revealed that they were closely correlated with extracellular matrix, extracellular space and ECM structuration.

Previous studies demonstrated that *LOX* and *LOXL2* were over‐expressed in KIRC.^33^, ^34^ *LOX* was reported to promote tumor progression and distant metastasis via enhancing matrix stiffness in KIRC.^34^ Upregulated *LOXL2* was also significantly correlated with higher pathological stages, cancer cell survival, invasion, and metastasis in KIRC.^34^ Recent studies have shown that the tumor microenvironment was closely related to the prognosis of tumor patients. *LOXL2* was reported to play pivotal roles in the formation of tumor microenvironment and metastatic niche in hepatocellular carcinoma.^35^, ^36^ *LOXL2* derived by cancer‐associated fibroblasts has also been confirmed to be an important mediator of intercellular communication in prostate tumor.^37^ In our study, we also found that the expression of *LOX* and *LOXL2* were significantly correlated with immune infiltration of immune cells (eg, CD8+ T cell, Macrophage, Neutrophil, and Dendritic cell). Based on these findings, we hypothesized that remolding the tumor microenvironment via blocking *LOXL2* may be an effective treatment for KIRC.

It is undeniable that there are some limitations in our research. First of all, our results were all based on database analysis, and lack further experimental confirmation. In our future research, we need to construct cell lines that differently expressed *LOX* and *LOXL2* to verify our research results from the aspects of in vivo, in vitro and KIRC tumor tissue. Secondly, our analysis indicated that *CP* and *PLOD2*, *COL1A2* were co‐expressed genes of *LOX* and *LOXL2*, respectively. However, how they cooperate with *LOX* and *LOXL2* in KIRC are unclear. Whether they can be used as gene panels in the diagnosis and treatment of KIRC also require further research.

In summary, we have confirmed the upregulation of *LOX* and *LOXL2* in KIRC, and further validated prognostic significance of *LOX*, *LOXL2* and their co‐expressed genes in KIRC. We hypothesize that these differently expressed genes may be promising molecular targets for the early diagnosis and targeted therapy of KIRC.

## CONFLICT OF INTEREST

The author(s) declare no competing interests.

## AUTHORS’ CONTRIBUTION

Zheng Liu was responsible for the study concept and design; Shitong Lin and Lingling Zheng were involved in data collection, data screening, statistical analysis, and wrote the manuscript. Other authors modified and took charge of supervising the manuscript. The final manuscript was approved by all the authors above.

## Supporting information

Fig S1

Fig S2

## Data Availability

Not applicable.

## References

[1] Hsieh JJ , Purdue MP , Signoretti S , et al. Renal cell carcinoma. Nature Rev Dis Primers. 2017;3(1):17009. 28276433 10.1038/nrdp.2017.9 PMC5936048

[2] Bray F , Ferlay J , Soerjomataram I , Siegel RL , Torre LA , Jemal A . Global cancer statistics 2018: GLOBOCAN estimates of incidence and mortality worldwide for 36 cancers in 185 countries. CA Cancer J Clin. 2018;68(6):394‐424. 30207593 10.3322/caac.21492

[3] Shuch B , Amin A , Armstrong AJ , et al. Understanding pathologic variants of renal cell carcinoma: distilling therapeutic opportunities from biologic complexity. Eur Urol. 2015;67(1):85‐97. 24857407 10.1016/j.eururo.2014.04.029

[4] Ravaud A , Motzer RJ , Pandha HS , et al. Adjuvant sunitinib in high‐risk renal‐cell carcinoma after nephrectomy. N Engl J Med. 2016;375(23):2246‐2254. 27718781 10.1056/NEJMoa1611406

[5] Gao H , Li Y , Lin T , Cheng Y , Ma Y . CIP2A promotes the survival of renal clear cell carcinoma Caki‐2 cells. FEBS open bio. 2020. 10.1002/2211-5463.12870 PMC9533430 32339416

[6] Kumar R , Kapoor A . Current management of metastatic renal cell carcinoma: evolving new therapies. Curr Opinion Supportive Palliative Care. 2017;11(3):231‐237. 10.1097/SPC.0000000000000277 28590313

[7] Kotecha RR , Voss MH . Systemic therapy for metastatic renal cell carcinoma‐is timing everything? Annals Transl Med. 2019;7(Suppl 6):S185. 10.21037/atm.2019.07.46 PMC6789351 31656764

[8] Wang SX , Mure M , Medzihradszky KF , et al. A crosslinked cofactor in lysyl oxidase: redox function for amino acid side chains. Science. 1996;273(5278):1078‐1084. 8688089 10.1126/science.273.5278.1078

[9] Smith‐Mungo LI , Kagan HM . Lysyl oxidase: properties, regulation and multiple functions in biology. Matrix Biol. 1998;16(7):387‐398. 9524359 10.1016/s0945-053x(98)90012-9

[10] Kim Y , Roh S , Park JY , Kim Y , Cho DH , Kim JC . Differential expression of the LOX family genes in human colorectal adenocarcinomas. Oncol Rep. 2009;22(4):799‐804. 19724858 10.3892/or_00000502

[11] Kim Y , Boyd CD , Csiszar K . A new gene with sequence and structural similarity to the gene encoding human lysyl oxidase. J Biol Chem. 1995;270(13):7176‐7182. 7706256 10.1074/jbc.270.13.7176

[12] Maki JM , Kivirikko KI . Cloning and characterization of a fourth human lysyl oxidase isoenzyme. Biochem J. 2001;355(Pt 2):381‐387. 11284725 10.1042/0264-6021:3550381 PMC1221749

[13] Asuncion L , Fogelgren B , Fong K , Fong S , Kim Y , Csiszar K . A novel human lysyl oxidase‐like gene (LOXL4) on chromosome 10q24 has an altered scavenger receptor cysteine rich domain. Matrix Biol. 2001;20(7):487–491. 11691588 10.1016/s0945-053x(01)00161-5

[14] Claude Jourdan‐Le Saux HT , Bogic L , Bryant‐Greenwood GD , Boyd CD , Csiszar K . The LOXL2 gene encodes a new lysyl oxidase‐like protein and is expressed at high levels in reproductive tissues. J Biol Chem. 1999;274(18):12939‐12944. 10212285 10.1074/jbc.274.18.12939

[15] Kasashima H , Yashiro M , Okuno T , et al. Significance of the lysyl oxidase members lysyl oxidase like 1, 3, and 4 in gastric cancer. Digestion. 2018;98(4):238‐248. 30045039 10.1159/000489558

[16] Cao C , Lin S , Zhi W , et al. LOXL2 expression status is correlated with molecular characterizations of cervical carcinoma and associated with poor cancer survival via epithelial‐mesenchymal transition (EMT) phenotype. Front Oncol. 2020;10:284. 32211324 10.3389/fonc.2020.00284 PMC7067748

[17] Kim YH , Goh TS , Heo H , Han M‐E , Jeong D , Lee C‐S , et al. ESurv: a user‐friendly online integrative survival analysis tool (Preprint); 2019 10.2196/preprints.16084

[18] Rhodes DR , Yu J , Shanker K , et al. ONCOMINE: a cancer microarray database and integrated data‐mining platform. Neoplasia. 2004;6(1):1‐6. 15068665 10.1016/s1476-5586(04)80047-2 PMC1635162

[19] Tang Z , Li C , Kang B , et al. GEPIA: a web server for cancer and normal gene expression profiling and interactive analyses. Nucleic Acids Res. 2017;45(W1:W98‐W102. 28407145 10.1093/nar/gkx247 PMC5570223

[20] Chandrashekar DS , Bashel B , Balasubramanya SAH , et al. UALCAN: a portal for facilitating tumor subgroup gene expression and survival analyses. Neoplasia. 2017;19(8):649‐658. 28732212 10.1016/j.neo.2017.05.002 PMC5516091

[21] Cerami E , Gao J , Dogrusoz U , et al. The cBio cancer genomics portal: an open platform for exploring multidimensional cancer genomics data. Cancer Discov. 2012;2(5):401‐404. 22588877 10.1158/2159-8290.CD-12-0095 PMC3956037

[22] Li T , Fan J , Wang B , et al. TIMER: a web server for comprehensive analysis of tumor‐infiltrating immune cells. Can Res. 2017;77(21):e108‐e110. 10.1158/0008-5472.CAN-17-0307 PMC6042652 29092952

[23] Li BO , Severson E , Pignon J‐C , et al. Comprehensive analyses of tumor immunity: implications for cancer immunotherapy. Genome Biol. 2016;17(1):174. 27549193 10.1186/s13059-016-1028-7 PMC4993001

[24] Zhou Y , Zhou B , Pache L , et al. Metascape provides a biologist‐oriented resource for the analysis of systems‐level datasets. Nat Comm. 2019;10(1):1523. 10.1038/s41467-019-09234-6 PMC6447622 30944313

[25] Smoot ME , Ono K , Ruscheinski J , Wang PL , Ideker T . Cytoscape 2.8: new features for data integration and network visualization. Bioinformatics. 2011;27(3):431‐432. 21149340 10.1093/bioinformatics/btq675 PMC3031041

[26] Uhlen M , Fagerberg L , Hallstrom BM , et al. Proteomics. Tissue‐based map of the human proteome. Science. 2015;347(6220):1260419. 25613900 10.1126/science.1260419

[27] Liu J , Lichtenberg T , Hoadley KA , et al. An integrated TCGA pan‐cancer clinical data resource to drive high‐quality survival outcome analytics. Cell. 2018;173(2):400‐16.e11. 29625055 10.1016/j.cell.2018.02.052 PMC6066282

[28] MacLennan S , Imamura M , Lapitan MC , et al. Systematic review of perioperative and quality‐of‐life outcomes following surgical management of localised renal cancer. Eur Urol. 2012;62(6):1097‐1117. 22841673 10.1016/j.eururo.2012.07.028

[29] Cui H , Shan H , Miao MZ , et al. Identification of the key genes and pathways involved in the tumorigenesis and prognosis of kidney renal clear cell carcinoma. Sci Rep. 2020;10(1). 10.1038/s41598-020-61162-4 PMC7060270 32144299

[30] Wang Q , Zhang H , Chen Q , Wan Z , Gao X , Qian W . Identification of METTL14 in kidney renal clear cell carcinoma using bioinformatics analysis. Dis Markers. 2019;2019(5648783):1–11. 10.1155/2019/5648783 PMC6954481 31976022

[31] Gonzalez‐Avila G , Sommer B , Garcia‐Hernandez AA , Ramos C . Matrix metalloproteinases' role in tumor microenvironment. Adv Exp Med Biol. 2020;1245:97‐131. 32266655 10.1007/978-3-030-40146-7_5

[32] Rigoglio NN , Rabelo ACS , Borghesi J , et al. The Tumor Microenvironment: Focus on Extracellular Matrix. Adv Exp Med Biol. 2020;1245:1‐38. 32266651 10.1007/978-3-030-40146-7_1

[33] Di Stefano V , Torsello B , Bianchi C , et al. Major action of endogenous lysyl oxidase in clear cell renal cell carcinoma progression and collagen stiffness revealed by primary cell cultures. Am J Pathol. 2016;186(9):2473‐2485. 27449199 10.1016/j.ajpath.2016.05.019

[34] Hase H , Jingushi K , Ueda Y , et al. LOXL2 status correlates with tumor stage and regulates integrin levels to promote tumor progression in ccRCC. Mol Cancer Res. 2014;12(12):1807‐1817. 25092917 10.1158/1541-7786.MCR-14-0233

[35] Wong C‐L , Tse A‐W , Huang Y‐P , et al. Lysyl oxidase‐like 2 is critical to tumor microenvironment and metastatic niche formation in hepatocellular carcinoma. Hepatology. 2014;60(5):1645‐1658. 25048396 10.1002/hep.27320

[36] Wang M , Zhao X , Zhu D , et al. HIF‐1α promoted vasculogenic mimicry formation in hepatocellular carcinoma through LOXL2 up‐regulation in hypoxic tumor microenvironment. J Exp Clin Cancer Res. 2017;36(1):60. 28449718 10.1186/s13046-017-0533-1 PMC5408450

[37] Nguyen EV , Pereira BA , Lawrence MG , et al. Proteomic profiling of human prostate cancer‐associated fibroblasts (CAF) reveals LOXL2‐dependent regulation of the tumor microenvironment. Mol Cellular Proteomics. 2019;18(7):1410‐1427. 10.1074/mcp.RA119.001496 PMC6601211 31061140

