# Supplementary figures and images for: Comprehensive analysis on the expression levels and prognostic values of LOX family genes in kidney renal clear cell carcinoma
Source: Cancer Med. 2020 Sep 24;9(22):8624–38. doi: 10.1002/cam4.3472 (PMC7666732; doi:10.1002/cam4.3472)

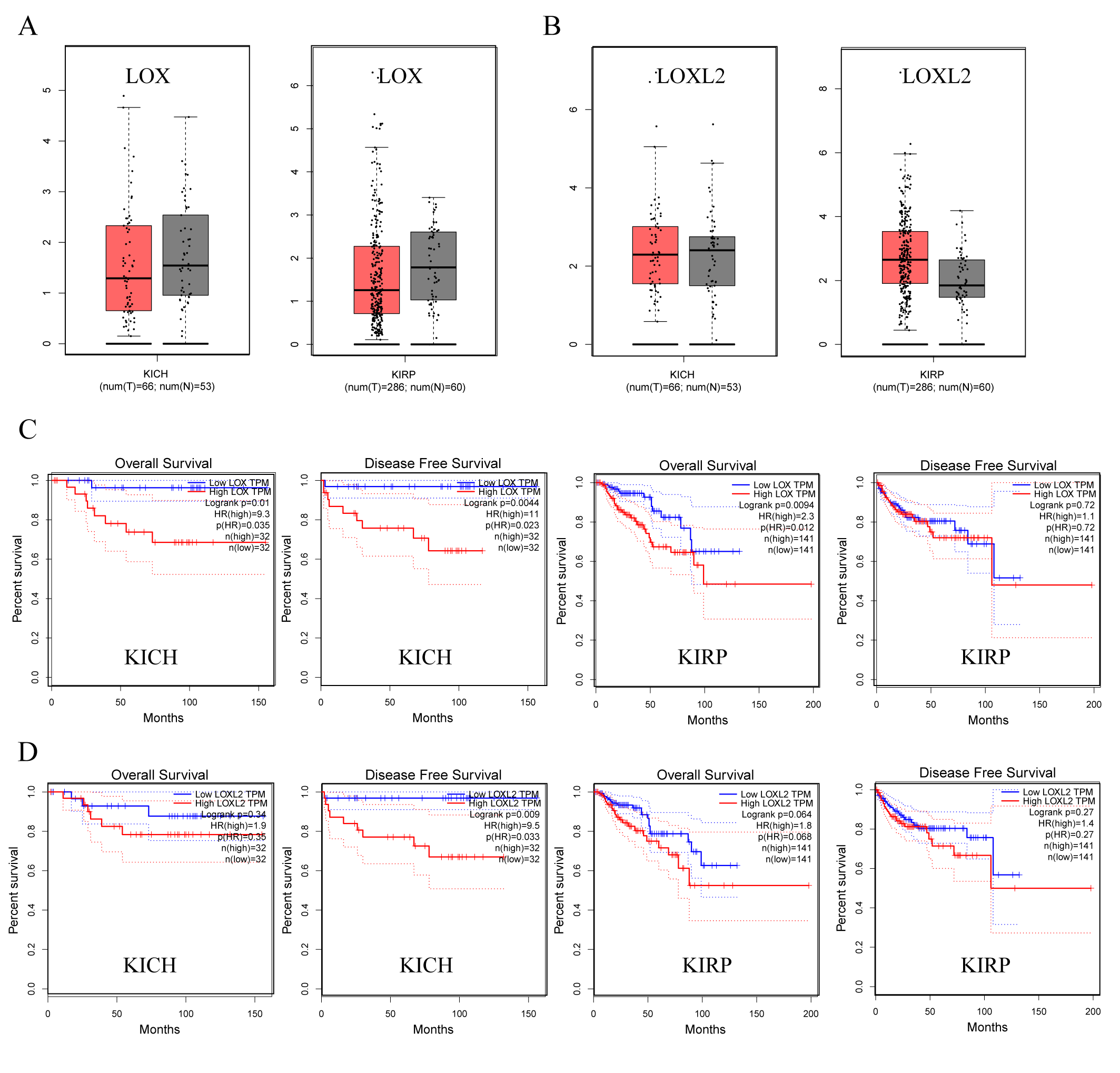

Supplement: Supplementary file 1 — Fig S1 [file CAM4-9-8624-s001.tif]

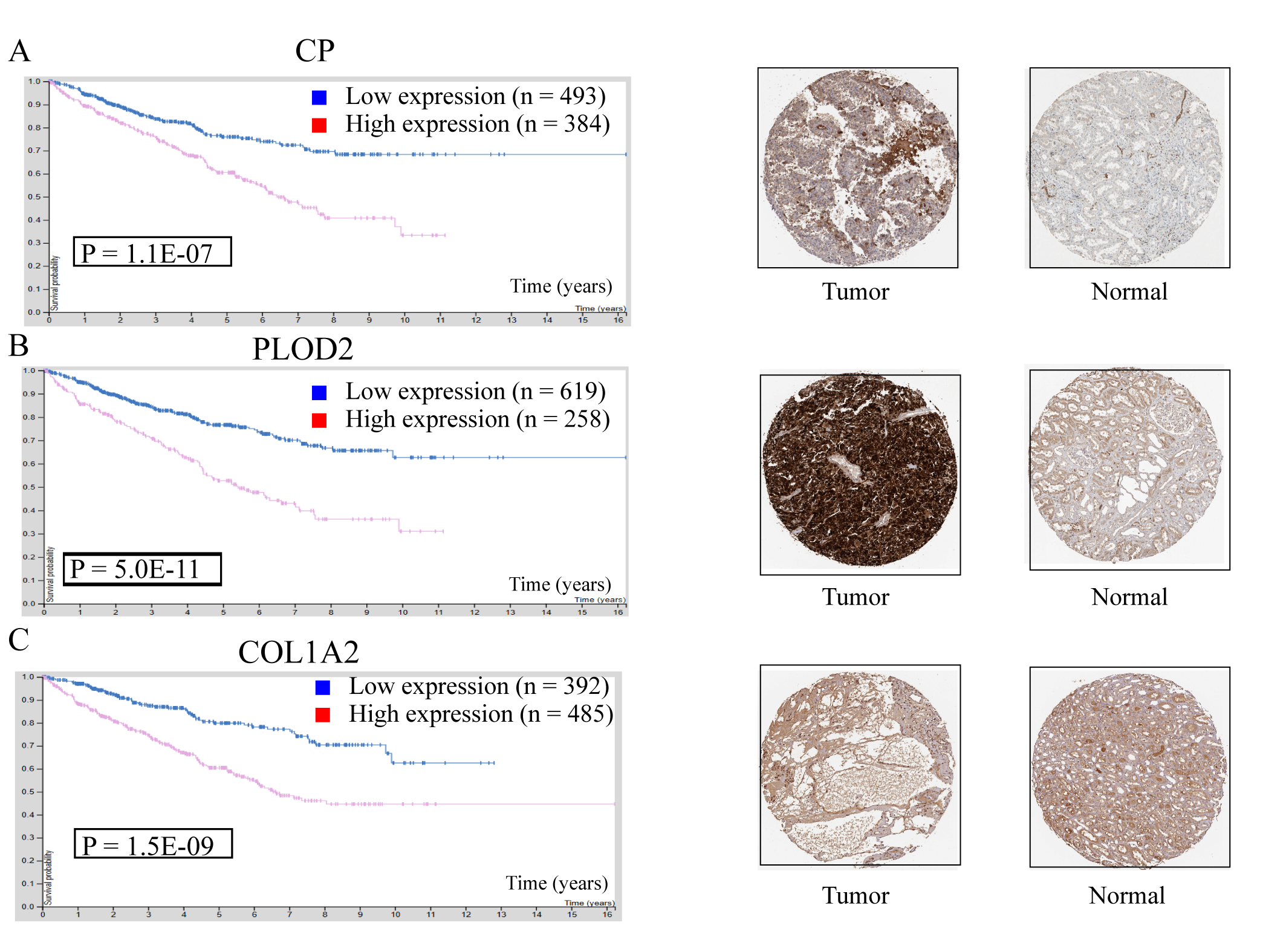

Supplement: Supplementary file 2 — Fig S2 [file CAM4-9-8624-s002.tif]
